# Intensive Training with Virtual Reality on Mobility in Adolescents with Cerebral Palsy—Single Subject Design

**DOI:** 10.3390/ijerph181910455

**Published:** 2021-10-05

**Authors:** Elisa Valenzuela, Renata Rosa, Carlos Monteiro, Leslie Keniston, Kênnea Ayupe, Jaqueline Frônio, Paula Chagas

**Affiliations:** 1 Graduate Program in Rehabilitation Sciences and Physical Functional Performance, Physical Therapy School, Universidade Federal de Juiz de Fora (UFJF), Juiz de Fora 36038-330, Brazil; elisajvalenzuela@yahoo.com.br (E.V.); jaqueline.fronio@ufjf.edu.br (J.F.); 2 Graduate Program in Reahabilitation Sciences, Medicine School, Universidade de São Paulo, São Paulo 01246-903, Brazil; renata.martinsr@hotmail.com (R.R.); carlosmonteiro@usp.br (C.M.); 3 School of Physical Therapy, Universidade Federal de Juiz de Fora (UFJF), Juiz de Fora 36036-330, Brazil; 4 Department of Physical Therapy, University of Maryland Eastern Shore (UMES), Princess Anne, MD 21613, USA; lpkeniston@umes.edu; 5 Physical Therapy School, Universidade de Brasília (UnB), Brasília 72220-275, Brazil; kennea.almeida@gmail.com

**Keywords:** adolescents, cerebral palsy, functioning, intensive training, virtual reality

## Abstract

Purpose: To evaluate the effects of a short-term intensive virtual reality intervention in adolescents with cerebral palsy (CP). Methods: Single-subject design, type A–B-follow-up, with four participants (P) with CP, 15–18 years, GMFCS level II. A two-week intervention phase was performed with twelve Nintendo^®^ Wii games in six sessions (90 min) per week. Outcome variables were semi-static balance (Pressure Center Oscillation—PCO), gait speed (Ten Meter Walk Test at usual speed—TMWT-U; and fast speed—TMWT-F), mobility (Timed Up and Go test—TUG), endurance (sit-to-stand test 5 times—STS-5), and gross motor activity (Gross Motor Function Measure—GMFM). Results: Statistical improvements were observed in GMFM-D (P2–P3), TMWT-F (P2–P3–P4) and TMWT-U (P2), STS-5 (P3–P4), TUG (P3), and PCO (P2–P3), assessed by level, trend, latency, and visual inspection to analyze change. Conclusions: This study shows that a short-term intensive intervention using Nintendo^®^ Wii-based games in adolescents, GMFCS level II, can be an effective therapy, leading to some recovery of functioning in these young people.

## 1. Introduction

The high prevalence of cerebral palsy (CP) in both child and adolescent populations [1,2], coupled with reduced performance in activities and social participation, demonstrates the need for studies that focus on ameliorating decreased functional outcomes in this population [3].

The use of recently developed technologies to provide treatments that are compatible with the CP population, such as virtual reality (VR) and its modalities, has grown in recent decades [4,5]. Virtual reality, as a compelling interactive tool, has some inherent advantages over other technologies and has demonstrated clinical effectiveness in improving functioning of children with CP [6,7,8,9,10]. Through an engaging environment, VR is a recommended therapy able to incorporate a variety of learning elements with interactive motor and cognitive challenges, offering opportunities for individuals with CP to participate in adaptive and challenging motor activities, which can expand their functional abilities [5].

Different possibilities for the use of VR have shown positive results, and the use of available non-immersive virtual reality video game consoles to provide “exergames” (i.e., a mixture of physical activity presented within the structure and flow of a game, which can function as a form of physical exercise) have been considered interesting [11,12]. According to Voinescu et al., in individuals with CP, the use of VR is a positive rehabilitation technique that provides movement, physical activity, and engagement [13]. Moreover, VR can provide improvement in lower limb strength, static and dynamic balance, mobility, increased gait speed, and improved gross motor function of the participants [6,7,8,9,10,14,15,16,17,18].

Studies have also shown that intensive VR practices in CP have grown in recent years, with faster, more effective results and with the possibility of maintaining functional gains for a longer time when compared to non-intensive intervention [5]. Despite this, there is still no consensus in the literature about the best frequency, intensity, and duration of intervention, making it difficult to draw uniform conclusions on this topic [6]. Thus, it is essential to develop studies, especially with the use of intensive VR in CP, in order to consolidate better intervention protocols in individuals with this health condition [6].

Another interesting gap is about the age of participants with CP in intensive VR protocols. Most studies have a mean age ranging from 6.9 to 9.6 years old, which may infer that the functional gains after an intervention protocol with VR are greater in children when compared to adolescents, and there are not many studies that have only participants in the second half of adolescence (15 to 19 years of age) [19,20]. To date, only a single study was found using virtual reality games exclusively in adolescent participants with PC [21].

Considering the above, the present study employed a non-immersive virtual reality exergame in an intensive protocol (two weeks, one and a half hours per day) in CP teenagers classified as Gross Motor Function Classification System (GMFCS) level II [22]. Participants were assessed using different motor scales: Gross Motor Function Measure (McKeith Press, London, UK), Mobility measures - 10-m walk test (TMWT), Timed-Up and Go-test (TUG), and Sit and Stand test—five times (STS-5); and Pressure Center Oscillation measure (M.P.S. Platform^®^, LorAnEngineering, Bologna, Italy) before and after the intervention. The hypothesis was that participants would present improved motor function after the intervention protocol. If this hypothesis is confirmed, the results will be relevant to the current literature, increasing the therapeutic possibilities through the use of an intensive virtual reality protocol in individuals with CP in the second half of adolescence.

## 2. Materials and Methods

### 2.1. Participants

Participants were selected by convenience, based on disclosure from Rehabilitation Services in a city in Minas Gerais, Brazil. Seven subjects were invited to participate, of which four accepted and met the following inclusion criteria: having a clinical diagnosis of CP, being between 15 and 18 years of age, and being at level II on the GMFCS. The adolescents were evaluated using the Mini Mental State Examination test (MMSE), and those with a cut-off score greater than 24 points were considered capable of understanding the simple orders necessary to perform the intervention protocol [23]. This study was approved by the institution’s Research Ethics Committee (CAAE: 82345718.6.0000.514). All participants and parents gave informed consent through signing the Informed Consent Form when they agreed to participate in the study.

### 2.2. Study Design

This was a single-subject design study, type A–B-follow-up, where one of the two trained researchers carried out the assessments in the three phases: (A) baseline, lasting two weeks, with four assessments carried out (two assessments each week); (B) intervention, lasting two weeks, with four assessments (two assessments each week); and (C) follow-up, lasting four weeks, with the removal of the intervention phase, with four assessments (one assessment each week) [24]. This study design has been considered to have a high level of evidence by the Oxford center for evidence-based medicine [25].

### 2.3. Setting and Interventions

**Figure 1:**
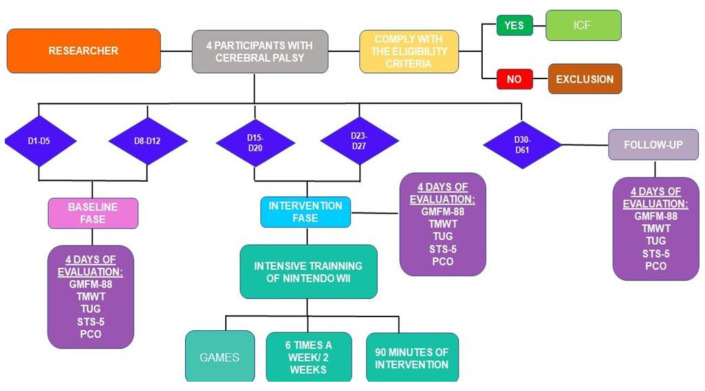
Study flowchart. Legend: Gross Motor Function Measure (GMFM-88), 10-m walk test (TMWT); Timed Up and Go (TUG); Sit to Stand Five times (STS-5); Pressure Center Oscillation (PCO); Informed Consent Form (ICF), Day 1–Day 5 (D1–D5); Day 8–Day 12 (D8–12); Day 15–Day 20 (D15–20); Day 23–Day 27 (D23–27); Day 30–Day 61 (D30–61).

The design and procedures of this study are described in Figure 1.

The researchers were composed of a physiotherapist responsible for carrying out the virtual reality intervention protocol with the Nintendo^®^ Wii, who was blind to the results of the assessments, plus two physiotherapists who carried out the assessments independently in all phases of the study, with an intra-class correlation coefficient (ICC) ≥ 0.99. The intervention protocol was carried out at the Child Performance Assessment Laboratory inside the School of Physical Therapy building.

### 2.4. Materials and Video Games

The intervention began in the second phase of the study (B) using a Nintendo^®^ Wii and Balance Board^®^ platform (Nintendo of America Inc., Redmond, USA) as resources. Each participant was given 15 min for familiarization with the console and the games used in the protocol at the end of the baseline phase (A). The frequency of the intervention was six times a week (from Monday to Saturday), for two weeks. On each intervention day, there were 6 gaming sessions lasting 15 min each, totaling 90 min. Participants were allowed to rest between gaming sessions (<=15 min). The duration of each game and the rest time were timed. In total, across the 2-week intervention period, 12 sessions were held, giving a total intervention time of 1080 min (18 h).

During each gaming session, the player performed all games in a standing position on the Balance Board^®^. During the intervention, the television was placed 2.5 m from the participants who were positioned between a hammock located in front of them and a wall (behind), so that in cases of imbalance, they could support themselves. The researcher was positioned next to the participants during the intervention, to provide security and support in the games, if necessary. All participants were instructed to continue their usual physical activity routines in the A phase of the A–B-follow-up study. However, in the intervention phase (B), due to the high hourly load of the protocol with the games, the participants only performed the intervention with the Nintendo Wii games. In the follow-up phase, the participants stopped performing the intervention protocol with games and returned to their activities of daily living.

The researchers set up a game protocol that was determined by the following sequence: Mondays, Wednesdays, and Fridays the participants played six games (Soccer Heading, Ski Slalom, Table Tilt, Tight rope Walk, Hula Hoop, and Basic Run); Tuesdays, Thursdays, and Saturdays, six other games were played (Penguin Slide, Balance Bubble, Ski Jump, Step, Boxy, Snowboard Slalom). The games were selected to produce possible effects on the mobility domain as described by the International Classification of Functioning disability and health (ICF) (Supplementary—Table S1) [20].

Information on the four study participants, regarding their health condition and functioning status prior to the intervention, as well as contextual and personal information from their respective caregivers, was collected through a structured interview according to the ICF (Supplementary—Figures S1–S4).

### 2.5. Study Outcomes and Evaluation Instruments

#### 2.5.1. Assessment Tools

According to the ICF, the outcomes of mobility capacity as a result of the physiotherapeutic virtual reality intensive intervention protocol with the Nintendo^®^ Wii were investigated using different instruments [20]. The first instrument applied to evaluate mobility capacity, by one of the evaluators, was the “Gross Motor Function Measure” (GMFM-88), dimensions D and E, with the objective of measuring the gross motor activity of children with CP, while standing, walking, running, and jumping [26,27].

Subsequently, the “10-m walk test” (TMWT) was applied, at the usual speed (TMWT-U) and at fast speed (TMWT-F). This tool uses the walking speed to provide valid clinical information regarding the capacity to walk [28]. Next, the “Timed Up and Go” test (TUG) was used, which assesses the capacity to stand, walk, rotate, and sit again. The shorter the test execution time, the better the mobility capacity evaluated by the TUG [29]. The “Sit and Stand test—five times” (STS-5) was used to assess the capacity to move from a sitting to standing position [30]. Finally, the capacity to change the body’s center of gravity through the “Pressure Center Oscillation” (PCO) was assessed using the Pressure Modular System Platform^®^ (M.P.S. Platform^®^, LorAnEngineering, Bologna, Italy) [31].

#### 2.5.2. Target Outcomes

In this study, the GMFM-88, dimensions D and E, was used. The total test score at baseline (estimated by GMFM-66 items) and percentage scores of these dimensions (in each evaluation) were obtained using the Gross Motor Ability Estimator (GMAE-2) software [26,27]. The higher the total score in the test, the better the participant’s mobility [26,27].

In the TMWT, the participants walked along a corridor with a distance of 14 m, using the intermediate 10 m for speed calculation. The speed was calculated as the distance traveled divided by the time taken in seconds (m/s), and the final speed was the average of three repetitions of each evaluation of the participants. The higher the speed in the TMWT-U and TMWT-F, the better the capacity to walk [28].

The TUG test was performed as follows: The participants started the test sitting in an armless chair, with hips and knees flexed at approximately 90°, barefoot and supported on a smooth floor; after a command they were required to get up from the chair, walk for 3 m at their usual walking speed, go around a cone on the floor, return to the chair where they started the test, and sit down again [29]. The participants performed the test activity three consecutive times with a rest interval of one minute between repetitions. For the TUG result, the average of the time of each of the three repetitions was used [29].

The STS-5 test requires an armless chair with a smooth floor. The participants are barefoot and begin sitting, with their arms free [30]. Participants are then asked to get up and sit down five times as fast as possible. A shorter time taken is considered as better mobility capacity and can infer better muscle endurance. The participants were required to perform this task three consecutive times, with a rest interval of one minute between repetitions. The shorter the mean time of these repetitions, the better the capacity in this task [30].

In the PCO test, participants were assessed in both conditions, eyes open (EO) and eyes closed (EC), in the standing posture, barefoot, performing 5 repetitions with a total of 30 s. In addition, the participants were evaluated with their feet positioned freely (when walking was interrupted). To analyze the results, the means of the five repetitions for each condition (EO and EC) were considered. The lower the speed of the PCO, the better the capacity to change the body’s center of gravity [31].

### 2.6. Statistical Analysis

Descriptive analysis of data was performed using the Statistical Package for the Social Sciences (IBM SPSS^®^, ver.22, Armonk, NY, USA), calculating the mean and standard deviation for all variables used in the study. For each participant, assessment of outcome significance was evaluated by level, trend, and latency, where visual inspection will indicate if a change refers to gradual increases or decreases in the dependent variable across observations: (1) between the baseline (A) and intervention (B) phases; and (2) between the intervention (B) and follow-up phases.

In brief, results of the 12 assessments, for each instrument and participant, were plotted on a graph where the x-axis represents the phases A–B-follow-up in weeks and the y-axis represents the scores obtained by the assessment instruments. In each graph, two continuous horizontal lines were drawn above and below the average of the four assessments performed in the baseline phase (A), representing two band standard deviations above and below the mean (band of two standard deviations). The values of each of the intervention (B) and follow-up phases were then compared with the baseline values. Differences were analyzed according to visual inspection of the direction of the outcome’s variables across phases. The GraphPad Prism^®^ program (v.5, 2007, San Diego, CA, USA) was used to construct the graphs.

## 3. Results

Four adolescents with CP, GMFCS level II, and mean age 16.25 years (SD = 1.41), participated in the study from July to November 2018. Contextual and personal information on participants and their respective caregivers is presented in Table 1.

All participants completed all phases of the study. The results of the GMFM test dimensions D and E, TMWT-U, TMWT-F, TUG, STS-5, and COP EO, and EC were plotted for each of the participants, according to each outcome investigated.

### 3.1. GMFM Scores

**Figure 2:**
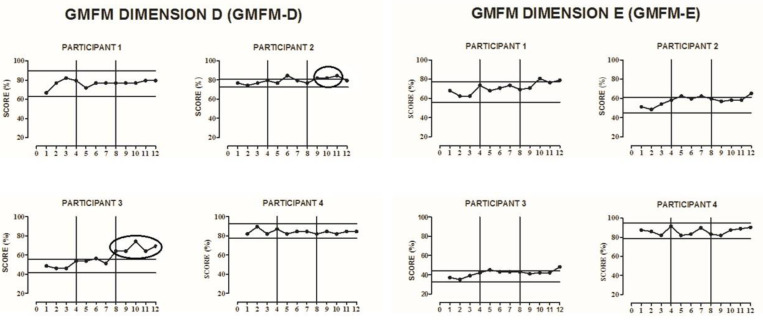
Visual representation of the Gross Motor Function Measure—Dimensions D and E at baseline, intervention, and follow-up phases for participants 1, 2, 3, and 4 (**P1**–**P4**). The solid horizontal lines indicate ±2 standard deviations from the mean of the baseline data. The x-axis represents the phases A–B-follow up, and the vertical lines divide the phases. The circles that encompass data show a change trend after a graphic visual inspection of the baseline.

For the GMFM dimension D and E scores, from the graphic visual analysis, trends of change were observed with an increase in dimension D scores in the participants P2 (from the 9th evaluation) and P3 (from the 8th evaluation) in the follow-up phase compared to the baseline (Figure 2). No differences were observed in dimension E in any of the participants (Figure 2).

### 3.2. Meter Walk Test

**Figure 3:**
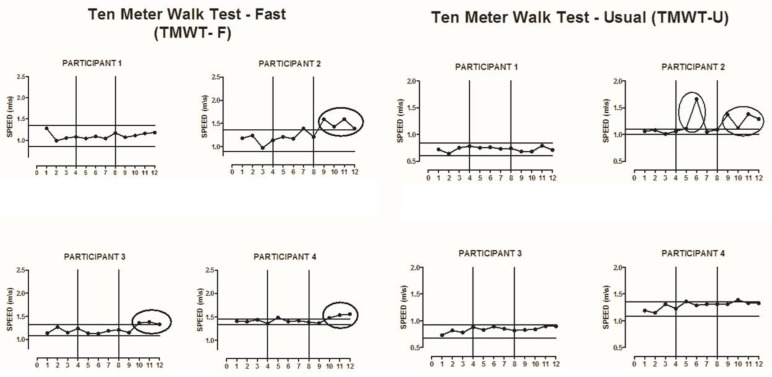
Visual representation of the Ten Meter Walk Test—Fast Speed (TMWT-F) and Ten Meter Walk Test—Usual Speed (TMWT-U) at baseline, intervention, and follow-up phases for participants 1, 2, 3, and 4 (**P1**–**P4**). The solid horizontal lines indicate ±2 standard deviations from the mean of the baseline data. The x-axis represents the phases A–B-follow up, and the vertical lines divide the phases. The circles that encompass data show a change trend after a graphic visual inspection of the baseline.

In the 10-m walk test, change trends were observed with increased walking speed in the TMWT-F in participants P2 (from the ninth evaluation), P3 (from the 10th evaluation), and P4 (from the 10th evaluation) in the follow-up phase (Figure 3) and a change trend in P2 (from the ninth assessment) in the TMWT-U (Figure 3).

### 3.3. Sit and Stand Test-Five Times

**Figure 4:**
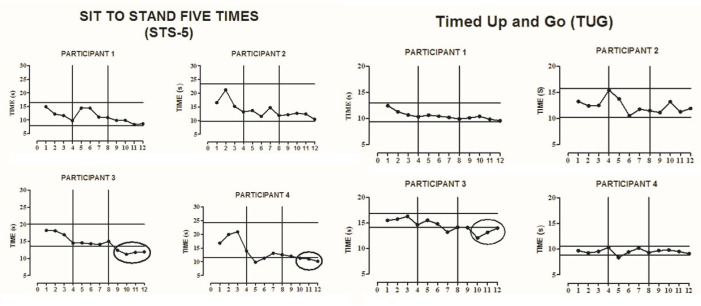
Visual representation of the Sit to Stand Five Times (STS-5) and Timed Up and Go (TUG) at baseline, intervention, and follow-up phases for participants 1, 2, 3, and 4 (**P1**–**P4**). The solid horizontal lines indicate ±2 standard deviations from the mean of the baseline data. The x-axis represents the phases A–B-follow up, and the vertical lines divide the phases. The circles that encompass data showed a change trend after a graphic visual inspection of the baseline.

From the graphical visual analysis, change trends were observed in the reductions in the time spent to perform the activity in the follow-up phase compared to baseline phase in two subjects (P3 and P4) in the ninth evaluation (Figure 4).

### 3.4. Timed Up and Go

After graphic visual inspection was observed, a decrease was noticed in the time spent to perform the activity in participant P3 (from the 10th evaluation), with no similar changes observed in the other participants (Figure 4).

### 3.5. Pressure Center Oscillation

**Figure 5:**
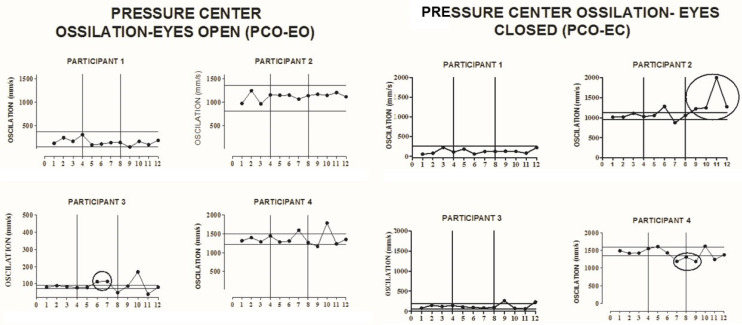
Visual representation of the Pressure Center Oscillation (PCO) with the Eyes Open (EO) and with the Eyes Closed (EC) at baseline, intervention, and follow-up phases for participants 1, 2, 3, and 4 (**P1**–**P4**). The solid horizontal lines indicate ±2 standard deviations from the mean of the baseline data. The x-axis represents the phases A–B-follow up, and the vertical lines divide the phases. The circles that encompass data showed a change trend after a graphic visual inspection of the baseline.

A change trend was found in the PCO with eyes open in participant P3 (in the sixth and seventh evaluations) (Figure 5), and with eyes closed in P2 (from the ninth evaluation) (Figure 4) with increased oscillation of the center of mass, which represents a worsening ability to change the center of mass. In P4 (from the seventh evaluation), there was a change trend in the PCO with eyes closed (Figure 5), with an improvement in the ability to change the center of mass at the end of the intervention phase.

## 4. Discussion

Adolescence has been recognized as an important time of transition, especially for individuals with CP, because many are thought to experience a decline in physical function during adolescence and early adulthood [32]. Given this, it becomes necessary to seek alternative interventions so that this population remains active and feels motivated to perform physical activities and achieve possible functional gains [32].

In view of this, the current study presents a new therapeutic possibility of virtual reality involving intensive use of a Nintendo^®^ Wii in the treatment of adolescents with CP. The current study showed that two weeks of intervention, 90 min a day, 6 days a week in adolescents with CP may possibly contribute to changes in mobility capacity, measured through different outcome instruments and incorporate possible gains from an intervention in their daily life activity.

Furthermore, this study adds to the current literature due to its differential of presenting an intensive intervention proposal with the use of video games for adolescents with different types of CP, since there is a lack of studies using this modality in this age group. In addition, the current study included only one type of functional classification, GMFCS level II, favoring the comparison of results between participants.

According to the results presented, change trends were found in the participants P2 (bilateral spastic) and P3 (bilateral ataxic). Both presented dysfunctions in mobility capacity to perform activities such as changing basic body position, maintaining a body position, transferring oneself, walking, and moving around. Both showed significant improvements in the GMFM dimension D test (standing) in the follow-up phase. The D dimension of the GMFM test assesses activities such as “standing on one foot” and “crouching”, which were performed by the participants during the intervention with Nintendo^®^ WII games [26,27]. It is important to note that none of the participants showed improvements in the GMFM-dimension E. The absence of changes in this dimension may be due to lack of intervention effects in this dimension, as it was a stationary training over the Nintendo^®^ Wii Platform or due to the motor limitations of adolescents GMFCS level II. Future studies should investigate these hypotheses.

As the change trend was observed in the follow-up phase, it can be assumed that these activities were incorporated into the participants’ daily routines after the intervention with the video game, allowing the emergence of new motor skills [28]. It is worth mentioning the importance of associating a virtual reality intervention with practices in activities that will promote the acquisition of motor skills in relevant daily activities [29].

The results of this study also showed change trends in the 10-m walk test at fast speed (TMWT-F) in participants P2 and P3 and in the 10-m walk test at usual speed (TMWT-U) in P2. This trend may have occurred due to the experience with the game “Basic Run”, where the objective of participants is to transfer weight from the left lower limb to the right lower limb in a synchronous and fast way. This experience may have facilitated an improvement in postural adjustment, semi-static and dynamic stability, and gait speed. This ability may also have been improved by the routine practice of the activity of “walking” in the daily life of the participants with CP in this study [33].

The current study corroborates a previous study that evaluated the effect of the Nintendo^®^ Wii on both children and adolescents with PC, in a clinical trial. In that study, the subjects were divided into two groups, with one group receiving both the intervention with the Nintendo^®^ Wii and treatment based on neurodevelopmental treatment. The other group received only the neurodevelopmental treatment [11]. The authors found significant improvements in gait speed, balance, postural control, muscle endurance, and mobility (*p* = 0.001) of the participants in the Wii group when compared to the control group [11]. These results corroborate our findings, in that participant P3 also showed significant changes in the STS-5 and TUG tests [11]. There is still no evidence in the literature highlighting increased muscle endurance assessed by the STS-5 test in children and adolescents with CP after interventions with a Nintendo^®^ Wii. Other studies need to be carried out with this measurement outcome to further affirm the results found in this study.

Participant P2 also demonstrated a change trend in the PCO with the eyes closed (EC). Two case studies have found decreased PCO oscillation in an adolescent of thirteen years, with bilateral CP, after eleven sessions with Nintendo^®^ Wii video games [12]. However, it was observed that participant P2 showed a change trend of an increase in the speed of oscillation with EC and P3 with EO. The observed trend in this participant was only present during the intervention phase and may represent a period of adjustment to the biomechanical disturbances occurring due to the Nintendo Wii protocol.

Participant P4 (bilateral dyskinetic) presented a change trend in function related to the mobility of the lower limb joints, strength, resistance, changes in muscle tone throughout the body, changes in the vestibular function of the movement, changes in fluency and rhythm of speech, and visual and auditory dysfunction. Participant (P4) did not present significant changes in the GMFM test dimensions D and E. His baseline did not stabilize in this outcome. Possibly this participant was having a testing-effect of the use of the GMFM, learning some of the items and this way showing a better score at each evaluation. In addition, he was the best one at this test, which probably impacted the absence of differences across phases.

Furthermore, P4 presented the highest performance in this test at the first evaluation (total GMFM-66 in the baseline = 72.6%), which may represent a ceiling effect in this test. In this case, the intervention with the Nintendo Wii improved the semi-static balance of the participant, with consequent benefits in gait performance.

Despite the considerable gains shown by most participants, it was observed that participant P1 (unilateral spastic) did not present any significant differences in any phase of the study. One possible explanation for this may be the specific impairments presented by subject P1. Having left unilateral CP, P1’s greatest impairment was in the upper limb. As this study’s protocol focused on games involving lower limb performance, it is conceivable that we were unable to explore potential improvements possible for those with upper limb dysfunction. As seen in several studies with specific protocols for upper limb dysfunctions (the main literature surrounding virtual reality protocols) [34,35], if participant P1 had performed a specific VR protocol for the upper extremities, significant improvements in function may have been attainable. This represents an excellent opportunity for a future investigation and one we plan to undertake soon.

Some barriers were identified during the data collection of this study, such as difficulty using public transport for caregivers and participants to arrive at the requested location; changing the schedules of the appointments of caregivers to accompany them during the phases of the study; the participants were attending school activities during the collection period, and often had to leave the school directly and go to the intervention; and the participants stopped performing their usual physical activities during phase B (intervention) of the study. Despite these barriers, there was strong attendance on the part of the participants and great family involvement.

Among the limitations of this study were the lack of stability during the baseline assessments (phase A), and the fact that we cannot completely assume that the positive effects observed in the follow-up phase were due exclusively to the intervention phase (B). In addition, this study design does not permit appropriate inferential analysis, making it necessary for future clinical trials to test the hypothesis raised with this study.

## 5. Conclusions

This study demonstrates that an intensive intervention using a Nintendo^®^ Wii in adolescents with different types of cerebral palsy could be a feasible, effective, and safe resource, bringing several benefits to the functioning of this population. Three of the four participants showed trends to promote improvements in mobility, demonstrating the incorporation of motor skills into activities of daily living. Despite the differences between the typology, this model of intervention seems reliable, but a larger and more homogeneous sample is needed.

The promising results of this study suggest that it is important to conduct more studies using intensive gaming in individuals with CP, especially including the use of VR protocols, both to validate the discovered benefits and to consolidate the best time, frequency, and duration of interventions in adolescents with CP and GMFCS level II.

## Figures and Tables

**Table 1:** Descriptive information on the participants and caregivers of the study.

ADOLESCENTS	P1	P2	P3	P4
Sex	F	M	M	M
Type	Unilateral spastic	Bilateral spastic	Bilateral ataxic	Bilateral dyskinetic
Age	15	15	18	17
Height	1.54 m	1.55 m	1.67 m	1.65 m
Weight	48 kg	45 kg	56 kg	48 kg
GMFCS	II	II	II	II
Mini mental	37	31	28	34
Adolescent Schooling	Incomplete elementary school	Incomplete elementary school	Incomplete high school	Incomplete high school
Interventions/Sports	Physiotherapy and aquatic therapy 2x/week	Physiotherapy 1x/week	Soccer 2x/week	Pilates 2x/week and soccer 1x/week.
Caregiver	**C1**	**C2**	**C3**	**C4**
Caregiver education	Complete High school	University graduation	Incomplete high school	Complete high school
Caregiver occupation	Accountant	Accountant	Home professional	Business woman

Legends: P—participants; F—female; M—male; GMFCS—Gross Motor Function Classification System; C—caregiver.

## Supplementary Materials

The following are available online at https://www.mdpi.com/article/10.3390/ijerph181910455/s1. Supplementary Table S1: Detailed information on the Nintendo wii^®^ games that were used in the protocol intervention study. Here we attempt to describe the goals of each game, the International Classification of Functioning (ICF) domains that were targeted, and the instruments used in the study to measure the associated outcomes. Figure S1: Cerebral Palsy–Unilateral. Figure S2: Cerebral Palsy–Bilateral. Figure S3: Cerebral Palsy–Ataxic. Figure S4: Cerebral Palsy–Discinetic.
