# Supplementary material for: Intensive Training with Virtual Reality on Mobility in Adolescents with Cerebral Palsy—Single Subject Design
Source: Int J Environ Res Public Health. 2021 Oct 5;18(19):10455. doi: 10.3390/ijerph181910455 (PMC8508310; doi:10.3390/ijerph181910455)
Supplement: Supplementary file 1 [file ijerph-18-10455-s001.zip › suppl.pdf]

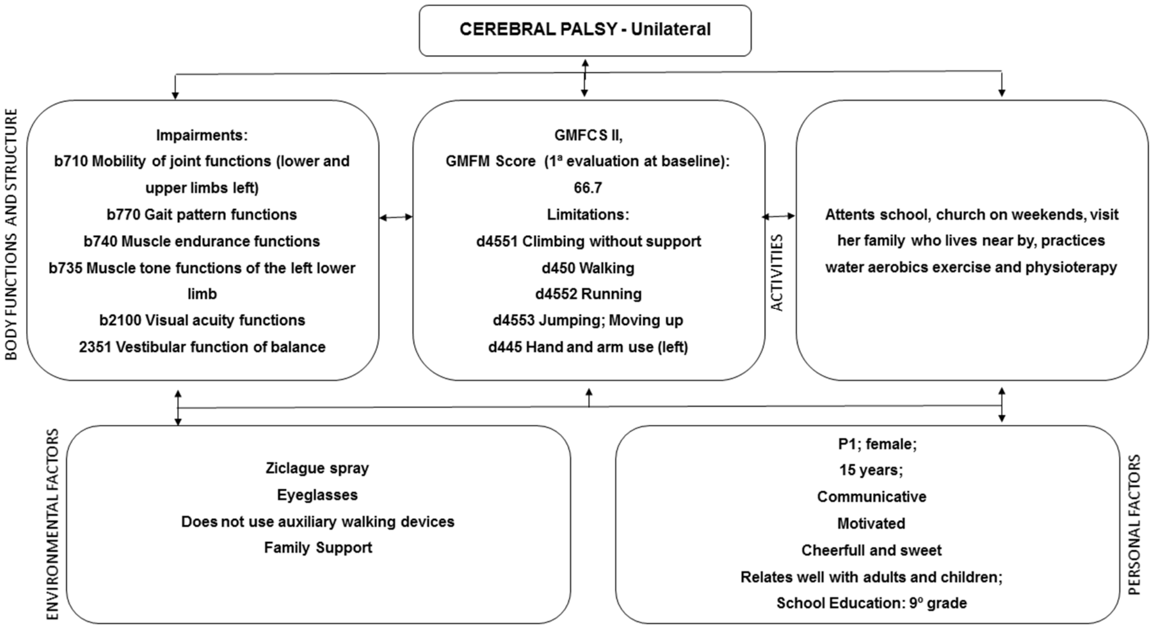

Figure S1. Cerebral Palsy – Unilateral.

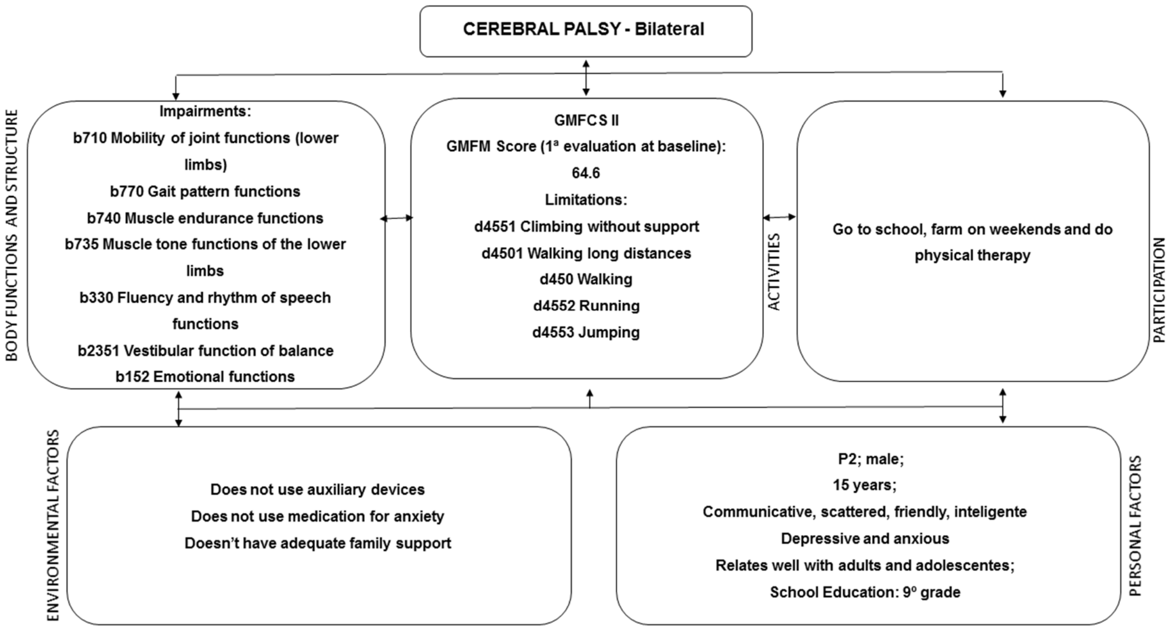

Figure S2. Cerebral Palsy – Bilateral.

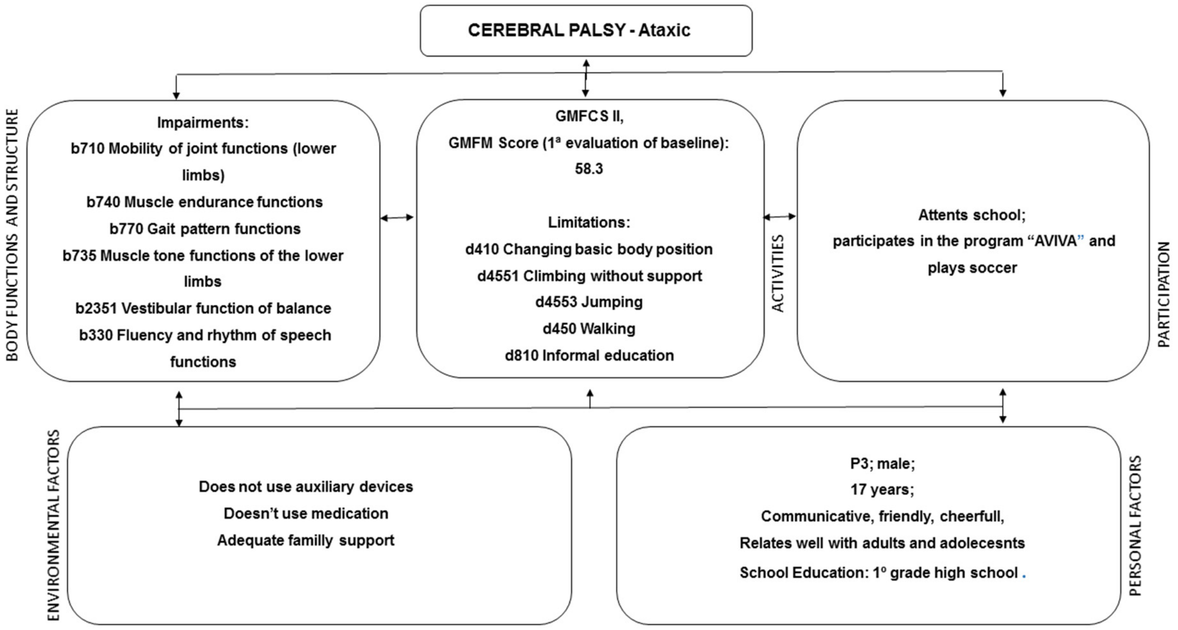

Figure S3. Cerebral Palsy – Ataxic.

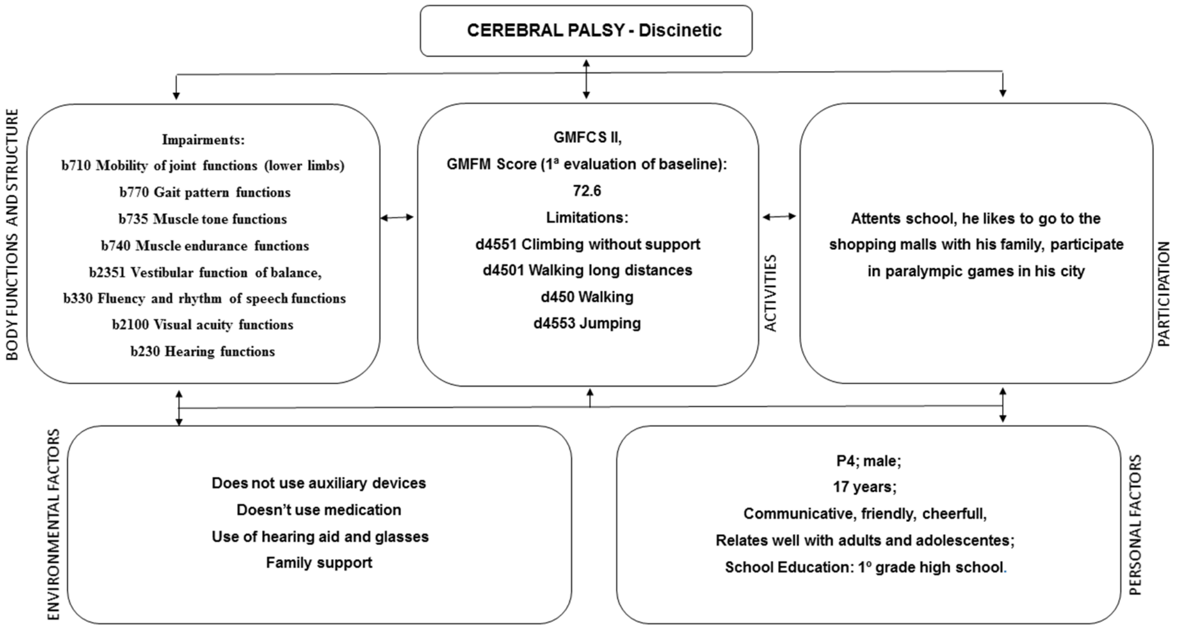

Figure S4. Cerebral Palsy – Discinetic.
