# Supplementary material for: Intensive Training with Virtual Reality on Mobility in Adolescents with Cerebral Palsy—Single Subject Design
Source: Int J Environ Res Public Health. 2021 Oct 5;18(19):10455. doi: 10.3390/ijerph181910455 (PMC8508310; doi:10.3390/ijerph181910455)
Supplement: Supplementary file 1 [file ijerph-18-10455-s001.zip › Table S1.pdf]

Table S1

Detailed information on the Nintendo wii® games that were used in the protocol intervention study. Here we attempt to describe the goals of each game, the International Classification of Functioning (ICF) domains that were targeted, and the instruments used in the study to measure the associated outcomes.

GAMES	GOALS	ICF	INSTRUMENTS
**SOCCER HEADING** 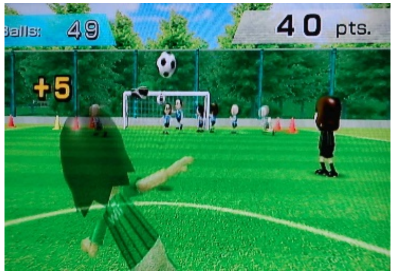	This game uses a soccer stadium scenario in which the objective of the game is for the participant to head balls in the goal by making lateral displacements of the lower limbs and pelvis. The more balls the player can head in the goal in one minute, the better their score in the game.	*Structures*: nervous system, eyes, ears, and structures of the lower extremities.<br>*Body Functions*: attention function, psychomotor function, perceptual functions, mental function of sequencing complex movements, seeing functions, hearing functions, vestibular functions, mobility and stability of joint functions, mobility of	*TUG*<br>*STS*-5<br>*GMFM*<br>PCO-EC<br>PCO-EO

	Improved scoring in this game also offers the player a chance to improve their ranking on a table of points, incentivizing performance in the game.	bone functions, muscle power function, muscle endurance function and muscle tone function.<br>*Activities:* Changing and maintaining basic body position, transferring oneself, moving objects with the lower extremity, bending, shifting the body's centre of gravity	
**SKI SLALOM**<br>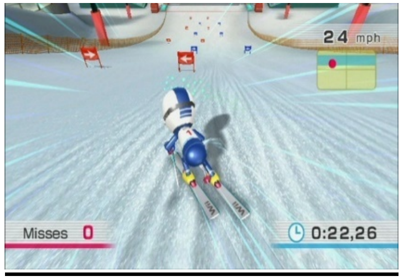	This game takes place on a ski slope, on which the participant must make lateral movements, squatting with the lower limbs, with the task of steering to pass between red	*Structure:* nervous system, eyes, ears, and structures of the lower extremities.<br>*Body Functions:* attention, psychomotor functioning, perceptual functions, mental functioning of	*TUG*<br>*STS-5*<br>*GMFM*<br>PCO-EC<br>PCO-EO

	and blue flags or go as fast as they can. The shorter the task's execution time, the better the performance. Similarly, the game offers being able to reach first place in a ranking of points with improved performance.	sequencing complex movements, seeing, hearing, vestibular, mobility, and stability of joints, mobility of bone functions, muscle power, muscle endurance and muscle tone functionality. *Activities:* Changing and maintaining basic body position, transferring oneself, moving objects with the lower extremities moving around, bending, shifting the body's centre of gravity and squatting.	
**TABLE TILT**	The game has the scenario placed on a board on which	*Structure*: of the nervous system, of the eyes, of the ear and structure of	*TUG*

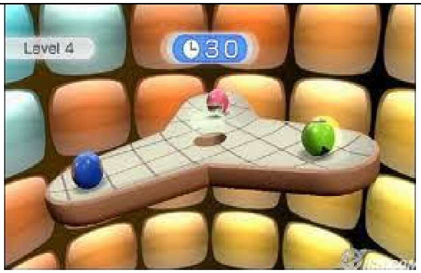	moves in response to the anteroposterior and laterolateral displacement of the player's lower limbs. The participant must aim and control as many of the colored balls to fall inside the hole in the center of the board in the initial forty seconds of gameplay. The faster the participant sinks the balls in the hole, they accumulate extra time to be used in the next stage of the game. Progression in the game is	lower extremity.<br>*Body Functions*: attention function, psychomotor function, perceptual functions, mental function of sequencing complex movements, seeing functions, hearing functions, vestibular functions, mobility and stability of joint functions, mobility of bone functions, muscle power, muscle endurance and muscle tone functionality.<br>*Activities*: Changing and maintaining basic body position, transferring oneself, moving objects with lower extremity moving around	*STS-5*<br>*GMFM*<br>PCO-EC<br>PCO-EO

	achieved by moving on to increasing degree of difficulty in each level (10 in total). A points table and ranking is also used.	and bending, shifting the body’s centre of gravity.	
**TIGHTROPE WALK**<br>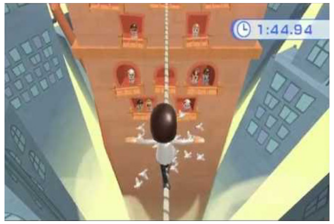	The game's scenario consists of a rope positioned between the roof of two buildings. The participant must alternately remove their feet from the Balance Board® platform so that they can move across the rope. They also must jump over obstacles that appear in the game by making	*Structure*: nervous system, eyes, ears and structures of lower extremities.<br>*Body Functions*: attention function, psychomotor function, perceptual functions, mental function of sequencing complex movements, seeing functions, hearing functions, vestibular functions, mobility and stability of joint functions, mobility of	*TUG*<br>*STS-5*<br>*GMFM*<br>PCO-EC<br>PCO-EO

	movements of squatting and standing up at appropriate times. The player has a time of two minutes to reach the other building, without losing balance and falling from the rope. The faster the player can perform the ropewalking task is rewarded by points scored and improved ranking in a table of points.	bone functions, muscle power function, muscle endurance function and muscle tone function. *Activities*: Changing and maintaining basic body position, transferring oneself, moving objects with lower extremity moving around, bending, shifting the body's centre of gravity and squatting.	
**HULA HOOP**	The game consists of a scenario where there is a "hula hoop" and the participant	*Structures*: nervous system, eyes, ears, and structures of the lower extremities, cardiovascular and	*TUG* *STS-5*

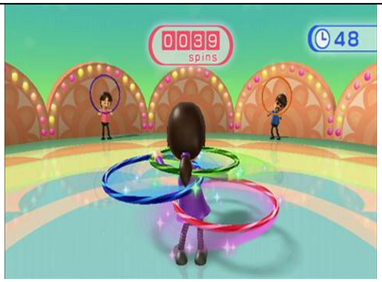	aims to make latero-lateral displacements with the lower limbs and rotational movements with the pelvic girdle, getting the hula hoop to rotate around the midriff as fast as they can. The faster the participant rotates the hula hoop during 1 minute of playtime, the higher their score and the better their performance in the game. A ranking points table is also present.	respiratory system.<br>*Body Functions*: attention, psychomotor functioning, perceptual functions, mental functioning of sequencing complex movements, seeing, hearing, vestibular, mobility, and stability of joints, mobility of bone functions, muscle power, muscle endurance, muscle tone functionality, heart and respiratory functioning.<br><u>Activities</u>: Changing and maintaining basic body position, transferring oneself, moving objects with lower extremity moving around,	*GMFM*<br>PCO-EC<br>PCO-EO

		bending, shifting the body's centre of gravity and squatting.	
**BASIC RUN**<br>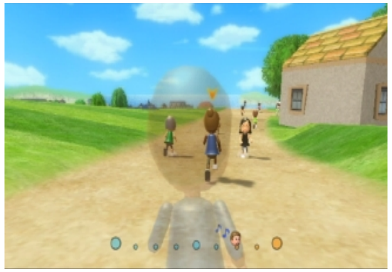	The game takes place in a park, in which the participant must simply run as fast as they can in three minutes playing time. The faster the player performs the task, the higher their energy expenditure will be. Similarly, an overall points table is employed.	*Structure*: nervous system, eyes, ears, and structures of the lower extremities, cardiovascular system and respiratory system.<br>*Body Functions*: attention, psychomotor functioning, perceptual functions, mental functioning of sequencing complex movements, seeing, hearing, vestibular, mobility, and stability of joints, mobility of bone functions, muscle power, muscle endurance and muscle tone	*TUG*<br>*STS-5*<br>*GMFM*<br>PCO-EC<br>PCO-EO

		functionality, heart, and respirations functionality<br>*Activities*: Changing and maintaining basic body position, transferring oneself, moving objects with lower extremity moving around, bending, shifting the body’s centre of gravity and running.	
**PENGUIN SLIDE**<br>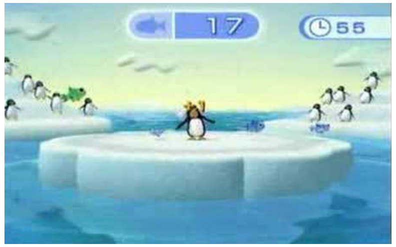 	The game's scenario consists of a penguin located on top of a block of ice. The participant must make latero-lateral and jumping movements, trying to capture the maximum number of fish that appear on the	Structure: nervous system, eyes, ears, and structures of the lower extremities.<br>*Body Functions*: attention, psychomotor functioning, perceptual functions, mental functioning of sequencing complex movements,	*TUG*<br>*STS-5*<br>*GMFM*<br>PCO-EC<br>PCO-EO

	screen. The faster the player performs the task of catching the fish, they will score higher in the game and can reach first place in the ranking of points.	seeing, hearing, vestibular, mobility, and stability of joints, mobility of bone functions, muscle power, muscle endurance and muscle tone functionality.<br>*Activities*: Changing and maintaining basic body position, transferring oneself, moving objects with lower extremity moving around, bending, shifting the body’s centre of gravity and squatting.	
**BALANCE BUBBLE**	The game scenario is a cartoon-like forest where the participant starts the game	Structure: nervous system, eyes, ears, and structures of the lower extremities.	*TUG*<br>*STS-5*

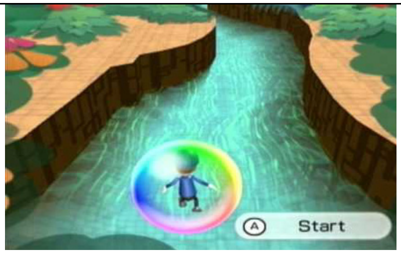	inside a bubble in a river. The goal is to traverse the entire route as fast as possible, without letting the bubble touch the side of the river and burst. For this, the player must make antero-posterior and latero-lateral displacements using the Balance Board® platform with the lower extremities to control the bubble. The faster you perform the task, the higher one can reach in the ranking of points.	*Body Functions*: attention, psychomotor functioning, perceptual functions, mental functioning of sequencing complex movements, seeing, hearing, vestibular, mobility, and stability of joints, mobility of bone functions, muscle power, muscle endurance and muscle tone functionality.<br><br><u>Activities</u>: Changing and maintaining basic body position, transferring oneself, moving objects with lower extremity moving around, bending, shifting the body's centre of gravity and squatting.	*GMFM*<br>PCO-EC<br>PCO-EO

**SKI JUMP** 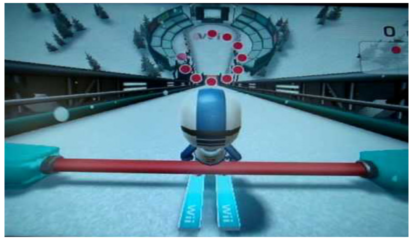	The scenario of the game is at an ski jumping hill, in which the participant starts at the top of the hill. Beginning a run, the player must perform an antero-posterior displacement of the lower limbs and crouch as far as they can to gain speed. Next, they will go down the hill with their knees bent and unload weight on the front of the foot. At an approaching red line, the player extends	*Structure*: of the nervous system, of the eyes, of the ear andstructure of lower extremity. *Body Functions*: attention function, psychomotor function, perceptual functions, mental function of sequencing complex movements, seeing functions, hearing functions, vestibular functions, mobility and stability of joint functions, mobility of bone functions, muscle power function, muscle endurance function and muscle tone function.	*TUG* *STS-5* *GMFM* PCO-EC PCO-EO

	their knees, jump and move the center of gravity of the body forward, thus “jumping” the greatest possible distance from the red line. The faster the task is performed, and the greater its displacement, the participant can rise in rank in the table ranking of points.	*Activities*: Changing and maintaining basic body position, transferring oneself, moving objects with lower extremity moving around, bending, shifting the body’s centre of gravity and squatting.	
**STEP**<br>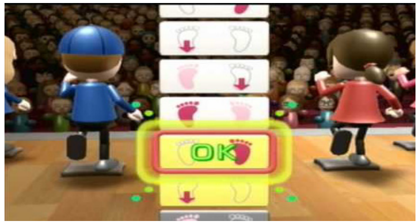 	The game takes place in a gym. The participant positions themselves with their feet on the ground, off the platform. When the game starts, images	*Structure*: nervous system, eyes, ears, and structures of the lower extremities.<br>*Body Functions*: attention, psychomotor functioning, perceptual	*TUG*<br>*STS-5*<br>*GMFM*<br>TMWT-F

	of a red foot will form on the screen, indicating the direction (right or left) in which the participant should step on the platform, alternately. The greater the number of correctly cued steps, the higher your score will be. Eventually, the participant can reach first place in the ranking of points.	functions, mental functioning of sequencing complex movements, seeing, hearing, vestibular, mobility, and stability of joints, mobility of bone functions, muscle power, muscle endurance and muscle tone functionality.<br>*Activities*: Changing and maintaining basic body position, transferring oneself, moving objects with lower extremity moving around, bending, shifting the body's centre of gravity and squatting.	TMWT-U<br>PCO-EC<br>PCO-EO

**BOXY**<br>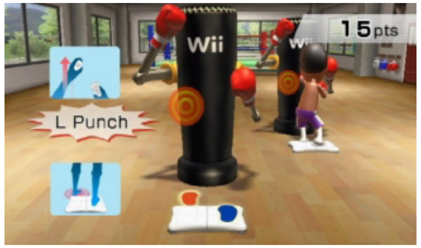	The scenario is a cartoon boxing training room, in which there is a teacher who says when and in what position (right or left) the participant must "punch" at the screen, alternating the upper and left and lower limbs right and left, together, When the participant performs the activity with the left upper limb, the left lower limb leaves the platform, touching the floor. The same will happen with the right upper limb and the right lower	*Structure:* nervous system, eyes, ears, and structures of the lower and upper extremities.<br>*Body Functions*: attention, psychomotor functioning, perceptual functions, mental functioning of sequencing complex movements, seeing, hearing, vestibular, mobility, and stability of joints, mobility of bone functions, muscle power, muscle endurance and muscle tone functionality, heart, and respirations functionality.<br>*Activities*: Changing and maintaining basic body position,	*TUG*<br>*STS-5*<br>*GMFM*<br>PCO-EC<br>PCO-EO

	limb. The more correctly cued actions the participant has, the higher their score.	transferring oneself, moving objects with lower extremity moving around, bending, shifting the body’s centre of gravity, squatting and fine hands use.	
**SNOWBOARD SLALOM**<br>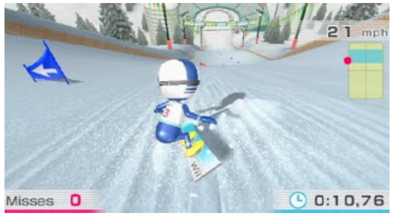	The scenario takes place in a ski slope where the participant must move laterally-laterally and antero-posteriorly to move in the game, in order to follow the arrows located on the blue flags, which will give the player's direction of travel (right, middle or left of the screen). The more correct	*Structure*: of the nervous system, of the eyes, of the ear andstructure of lower extremity.<br>*Body Functions*: attention function, psychomotor function, perceptual functions, mental function of sequencing complex movements, seeing functions, hearing functions, vestibular functions, mobility and stability of joint functions, mobility of	*TUG*<br>*STS-5*<br>*GMFM*<br>PCO-EC<br>PCO-EO

actions the participant executes in the less time, the higher the task will be his score. Thereby, the participant will be able to reach first place in the ranking of points.

bone functions, muscle power function, muscle endurance function and muscle tone function.

Activities: Changing and maintaining basic body position, transferring oneself, moving objects with lower extremity moving around, bending, shifting the body's centre of gravity and squatting.
